# Genetic Analysis of Clinical VZV Isolates Collected in China Reveals a More Homologous Profile

**DOI:** 10.1155/2013/681234

**Published:** 2013-05-27

**Authors:** Longfeng Jiang, Lin Gan, Jason Chen, Mingli Wang

**Affiliations:** ^1^Department of Microbiology, Anhui Medical University, Hefei 230032, China; ^2^Department of Pathology & Cell Biology, Columbia University, New York, NY 10032, USA

## Abstract

Forty-four varicella-zoster virus (VZV) isolates from China were genotyped by using a scattered single nucleotide polymorphism (SNP) method, including open reading frames (ORFs) 1, 22, 31, 37, 60, 62, 67, and 68. Based on the analysis of the polymorphic markers in the 8 ORFs, all of the 44 isolates can be placed in genotype J defined by the SNP profiles in ORF22 or clade B defined by the SNP profiles in ORFs 31, 37, 60, 62, 67, and 68. The three consecutive nucleotide (CGG) in-frame insertions in ORF 1 were found in 8 (18.2%) isolates, which has not been described in VZV strains from any other part of the world. A novel synonymous A>G substitution in ORF60 was revealed in 4 (9.1%) of the isolates. In addition, a previously described three consecutive nucleotide (ATC) insertion in ORF 60 was found in all the Chinese isolates but not in the US isolate MLS. The results showed all the 44 strains that belong to genotype J/clade B with significantly high homogeneity, and phylogenetic analysis suggested that the 44 Chinese isolates consist of 4 clusters, but interstrain variations also exist. Overall, VZV isolates obtained in China showed significantly higher genetic homogeneity than isolates reported from other countries.

## 1. Introduction

Varicella-zoster virus (VZV) is a member of the genus *Varicellovirus* within the subfamily of Alphaherpesvirinae, family of *Herpesviridae*. VZV is the etiologic agent of two diseases, varicalle (chickenpox) and zoster (shingles). Varicella occurs after the initial encounter with VZV, during which a lifelong latent infection is established. Latent virus can be reactivated, usually decades later in life, to cause zoster [1]. It is also a nearly ubiquitous pathogen that affects all human populations. However, the epidemiology of VZV infection varies geographically [2]. In temperate climate countries, most children have a primary VZV infection at school ages, whereas in tropical countries primary infection is often delayed until adulthood. In China, the epidemiology of varicella appears to be typical of temperate climate regions.

VZV is considered to have one of the most stable genomes of all herpesviruses. At the beginning, VZV was generally considered sufficiently stable to allow the use of a single sequenced virus (VZV-Dumas) as a consensual representation of the world VZV genotype, but later investigations have uncovered a gE mutant virus named VZV-MSP as the second genotype with a distinguishable accelerated cell spread phenotype [3]. By analysis of selected single nucleotide polymorphisms (SNPs) in ORFs 1, 21, 50, and 54, 4 genotypes were identified: A (Africa/Asia), B and C (Europe/North America), and J (Japan) in which B may represent recombinants between types A and C [4]. Loparev et al. combined ORF 22-based genotyping with the analysis of either ORF 21 or ORF 50 and described 5 confirmed clades E1, E2, J, M1, and M2 and the two provisional clades M3 and M4 [5, 6]. A third genotyping strategy was based on complete DNA sequences of 5 viral glycoprotein genes (gH, gI, gL, gB, and gE) and ORF62 major transactivator gene. Sequences obtained were clustered in 4 major clades, designated to A, B, C, and D [7, 8]. On the basis of SNPs in different ORFs, 5 major VZV clades (1–5) and two provisional clades (VI and VII) have been distinguished [9].

In this report, the genotyping scheme based on complete DNA sequences of five viral glycoprotein genes and the IE62 major transactivator gene or ORF22 was used to classify 44 different VZV isolates collected in Hefei, a city in the central Eastern part of China. In order to further evaluate the novel three nucleotide insertions in ORF1 we previously reported [10], the SNPs of ORF1 were also analyzed by sequencing. The results showed all the 44 strains that belong to genotype J/clade 2 with significantly high homogeneity, but interstrain variations also exist. 

## 2. Materials and Methods

### 2.1. Patients and Clinical Specimens

Forty-six specimens were obtained from 37 cases of herpes zoster and 9 cases of varicella. All of the patients (25 males and 21 females) were referred to the Dermatology Clinic of the First Affiliated Hospital of Anhui Medical University, Hefei, China, between July 2007 and July 2008. In all cases, vesicle fluid was collected in viral transport medium from skin lesions using a 1 mL hypodermic needle and was transported to the laboratory within 4 hours on wet ice [10]. Virus was recovered by inoculation of fluid from a single vesicle on human embryonic lung fibroblast (HELF) monolayer and was confirmed by immunofluorescence with anti-VZV gE monoclonal antibody (Biodesign International, ME, USA).

### 2.2. VZV DNA Extraction

Total DNA was purified from 200 *μ*L of virus infected HELF within 3 serial passages using a Takara MiniBEST Viral RNA/DNA Extraction Kit versis 3.0 (Takara Biotechnology Co. Ltd., Dalian, China) according to the manufacturer's instructions. The same method was used to extract DNA from HELF infected with the Oka vaccine strain (vOka), the parental Oka strain (pOka), and an American wild-type strain (MLS) (gifts from Dr. Anne A. Gershon at Columbia University). DNA from uninfected HELF cells was used as negative control in all experiments. The concentration of DNA was determined by spectrometry, and the DNA was stored at −20°C.

### 2.3. Genotyping and DNA Sequencing

The VZV genes ORF 31 (gB), ORF 68 (gE), ORF37 (gH), ORF 67 (gI), ORF 60 (gL), ORF 62 (IE62), ORF22, and ORF1 were amplified by polymerase chain reaction (PCR) with Pfu DNA polymerase (Promega, Madison, WI, USA) using corresponding oligonucleotide primers [5, 11]. For each pair of primers, a PCR negative control was included in which DNA extracted from uninfected HELF was used as the template. For detection of PCR products, 5 *μ*L of PCR products was loaded into 1% agarose gels and electrophoresed at 100 V for 30–60 min. Gels were stained with ethidium bromide and visualized under UV light. PCR products were purified with an Agarose Gel DNA Purification Kit (Takara Biotechnology, Dalian, China) and sequenced using BigDye Terminator Cycle Sequencing Kit (version 3.0; Applied Biosystems, UK) according to the manufacturer's instructions. Automated DNA sequencing was carried out with an ABI Prism 3730XL DNA Analyzer (Perkin-Elmer Applied Biosystems, Warrington, UK). Sequencing was performed with the PCR forward primers; however, reverse primers were employed to sequence the other strand whenever a different or an ambiguous nucleotide was found when it was compared with the published Dumas strain sequence (accession number NC_001348) in the homologous position.

### 2.4. Phylogenetic Analysis

Based on the analysis of the polymorphic markers in the 8 ORFs, all of the 44 isolates can be placed in genotype J defined by the SNP profiles in ORF22 [5, 6] or clade B defined by the SNP profiles in ORFs31, 37, 60, 62, 67, and 68 [7, 8]. The three consecutive nucleotide (CGG) in-frame insertions in ORF 1 were found in 8 (18.2%) isolates, which has not been described in VZV strains from any other part of the world. Concatenated sequences (ORFs1, 31, 37, 60, 62, 67, and 68) of China VZV isolates, the US wild-type MLS, and a few reference strains with different genotypes available from GenBank were aligned by MEGA 4.02 and used for phylogenetic analysis. The phylogenetic tree was verified using the neighbor joining (MEGA 4.02; model: Tamura-Nei; bootstrap analysis: 10,000 replicates) method.

## 3. Results

**Figure 1:**
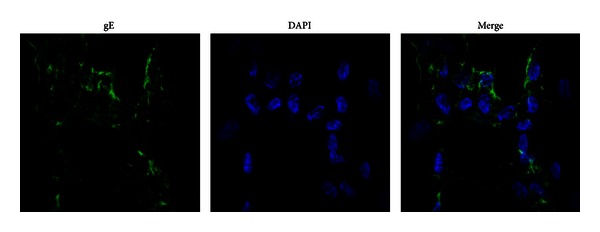
VZV specific antigen detected by indirect immunofluorescence using monoclonal antibodies against VZV glycoprotein E. Nuclei dyeing by DAPI and gE dyeing by fluorescein-conjugated secondary antibody.

Forty-four VZV isolates (95.7%) were obtained from 35 cases of herpes zoster and 9 cases of varicella and were sorted with Arabic numerals (Table 1). All isolates were identified by immunofluorescence with monoclonal antibodies to VZV glycoprotein E with green fluorescence in cytoplasm (Figure 1). 

**Figure 2:**
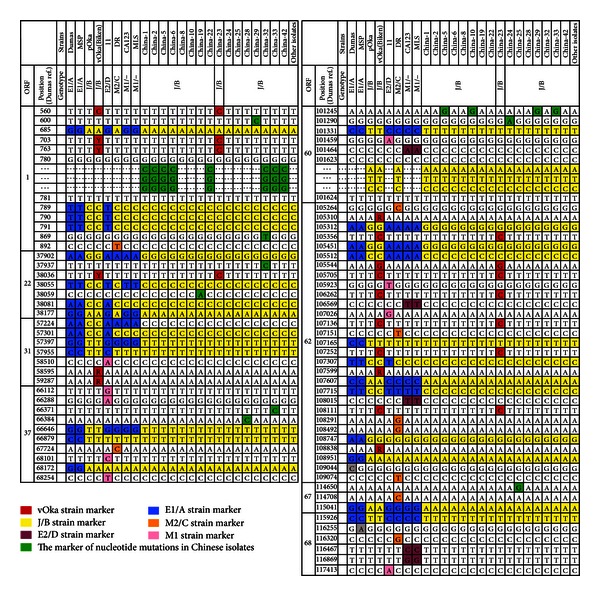
Genomic variations of 44 Chinese isolates. Sequence positions are based on the published genomic sequence of the Dumas strain (NC_001348). Cluster A markers are in blue, cluster B markers are in yellow, Oka vaccine markers are in red, and green represents markers unique to various single nucleotide mutations.

The sequencing results were aligned with reference sequences and assigned to corresponding genotypes in Figure 2. According to the nomenclature scheme proposed by Faga et al. [7], the 44 isolates could be assigned to clade B/clade 2 by analysis of SNPs in VZV ORFs 31, 37, 60, 62, 67, and 68 (Figure 2). The strains also fulfilled the definition of genotype J according to the SNP profiles in VZV ORF22 [5, 6] (Figure 2). We have previously reported a novel three consecutive nucleotide in-frame (CGG) insertion in ORF 1 between nucleotide positions 780 and 781 in the VZV genome [10]. Now we found this insertion was also present in new isolates, which count for 8 strains (18.2%) in total. A novel synonymous nucleotide substitution of A>G (SNP101245) in ORF 60 was identified in 4 isolates (9.1%) (Figure 2, China-5, -10, -29, and -33). Another 3 consecutive nucleotide (ATC) insertion in ORF 60 between nucleotide positions 101623 and 101624 was found in all the 44 isolates, which was reported in Singaporean and Thai isolates [12]. In addition, 12 single nucleotide substitutions were found in 5 of the 44 Chinese VZV isolates, China-8, -19, -25, -29, and -32, respectively. And all the SNPs analyzed for one isolate, China-23, were the same as those of vOka vaccine strain [13].

**Figure 3:**
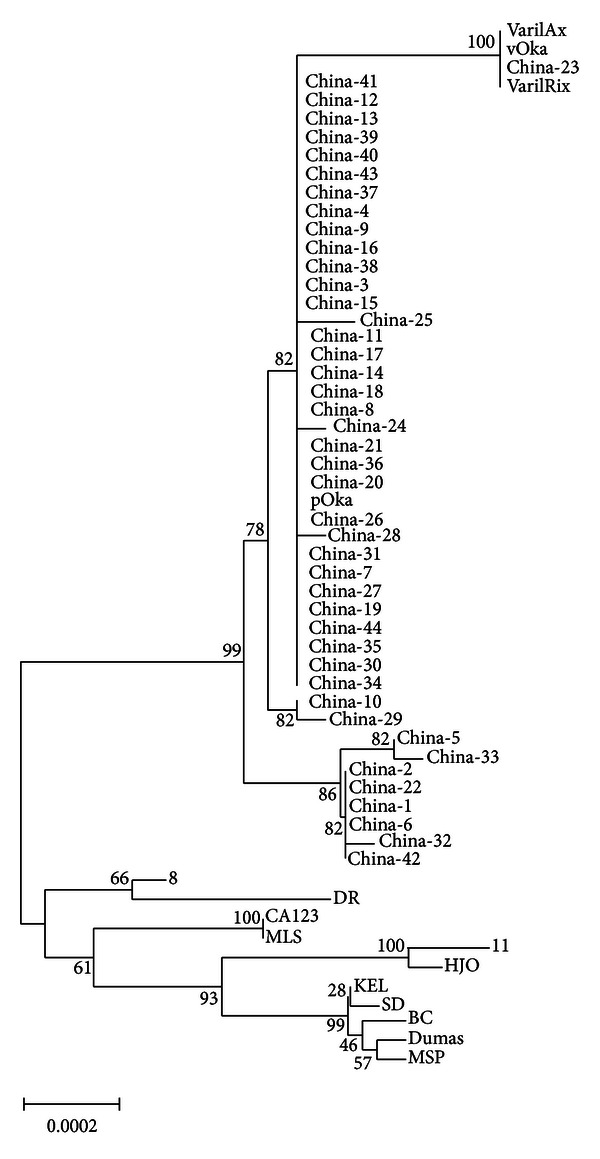
Phylogenetic tree of 44 VZV strains from patients with VZV infections and the reference strains as Dumas, KEL, SD, BC, MSP, pOka, vOka, VZV8, DR, VZV11, HJO, and CA123 based on DNA sequences in ORFs 1, 31, 37, 60, 62, 67, and 68.

The sequence data based on the ORFs1, 31, 37, 60, 62, 67, and 68 from the Chinese isolates were aligned with the published sequences from 9 reference strains that belong to different genotypes, and phylogenetic analysis was performed. The resulting tree is presented in Figure 3, and it revealed that the 44 Chinese wild-type isolates was distributed into 4 clusters. Six isolates (China-1, -2, -6, -22, -32, and -42) constituted the 1st cluster that contains a consecutive CGG insertion in ORF1. Two isolates (China-10, -29) formed the 2nd cluster and both of them have a synonymous nucleotide substitution of A>G in ORF60. The 3rd cluster may include two isolates (China-5, -33) and they have both the consecutive CGG insertion in ORF1 and the synonymous nucleotide substitution of A>G in ORF60. The remaining 33 Chinese isolates could be grouped into the 4th cluster which groups with the Japanese pOka strain.

## 4. Discussion

The genomic stability of VZV was well established, but limited genetic diversity with an intriguing correlation to geographic origin was documented [4, 5, 7]. The strain distribution may be driven by climate, host-virus interactions, immigration patterns, or other factors. In this study, based on the analysis of the polymorphic markers in 8 ORFs (ORFs 1, 22, 31, 37, 60, 62, 67, and 68), considerable homology was found among the 44 clinical strains. They may all be placed in clade B/genotype J/clade 2 based on different SNP profiles [5–9], but mutations also could be detected. The novel 3 nucleotide (CCG) insertion in ORF1 reported recently was found in a total of 8 (18.2%) of the 44 isolates. This insertion thus far has been found only in Chinese isolates but not in the US wild-type VZV strain that we analyzed (MLS strain in Figure 2). It was not present in all other strains that are available in GenBank including those of clade B such as pOka and vOka. Further investigation with strains from different continents is necessary to determine its distribution.

A novel SNP that resulted from the synonymous substitution of A>G (SNP101245) in ORF 60 was found in 9.1% of the Chinese isolates (Figure 2). VZV ORF60 encodes glycoprotein gL, which functions as a chaperone protein for gH protein [14], and no SNP was observed in ORF60 as yet [15]. There were two isolates that had both the 3-nucleotide insertion in ORF1 and the synonymous A>G substitution in ORF 60, and they constitute a cluster in the phylogenetic tree (China-5 and -33 in Figure 2). Another three consecutive nucleotide (ATC) insertion in ORF 60 between nucleotide numbers 101623 and 101624 was found in all 44 Chinese isolates, which had been found present in some Asia strains including those from Singapore, Bangkok, but not in any Western VZV strains [7, 8, 12]. The insertion results in addition of a methionine residue in gL that may potentially serve as an alternative start site. Our study suggested that this insertion may be commonly present in clade B/genotype J/clade 2 strains. It is of interest to note that this insertion was also found in DR strain, which was suggested as being a recombinant by genotypes E/clade1 and genotypes J/clade2 strains [16, 17].

It was previously reported that VZV strains with genotype E/clade1 and genotype M/clade 4 were identified in North and South China, respectively [5]; therefore, there are at least three genotypes of VZV circulating in China. It is our speculation that genotype J/clade 2 might be predominant in China, although studies involving strains from more places in China will be necessary before the conclusion is made. The vaccine strain found in this study was isolated from a 23-year-old woman with a mild case of varicella [13]. She had had close contact with a 5-year-old boy who developed shingles 13 months after vaccination with one dose of VarilRix. Since ORF62 is one of the largest VZV genes and encodes the major regulator of VZV transcription, it may contribute to the attenuation of vOka.

Phylogenetic analysis showed that all the 44 wild-type strains included in this study were highly homologous (Figure 3). This was not a surprise because the strains were isolated in Hefei, the capital of Anhui Province, where >99.9% residents are native Chinese. Nevertheless, genetic variations still exist in the strains that may be used as molecular markers in epidemiological surveillance. Four clusters may be differentiated based on the differences in SNPs and insertions. It likely represents private alleles specific to VZV populations within humans residing in localized geographic areas. Consistent with previous studies [7, 8, 17], our results support the concept that the history of the VZV evolution included recombination events. Ultimately, genome sequencing may prove to be the most useful tool for investigation of VZV transmission events. Continuous molecular epidemiologic surveillance of VZV strains circulating in the country may help to understand the current status of VZV infection in China.

## Figures and Tables

**Table 1:** Characteristics of VZV infections in Chinese patients from whom VZV strains were isolated between July 2007 and July 2008.

Clade	VZV isolate	Sex	Age (year)	Isolation	Patient type	Strain Origin	History of varicella
2	China-1	M	50	Yes	Ambulatory	Zoster	Childhood in China
2	China-2	M	45	Yes	Ambulatory	Zoster	Not available
2	China-3	M	70	Yes	Hospitalized	Zoster	Childhood in China
2	China-4	M	64	Yes	Ambulatory	Zoster	Childhood in China
2	China-5	M	34	Yes	Ambulatory	Zoster	Not available
2	China-6	M	67	Yes	Ambulatory	Zoster	Not available
2	China-7	M	35	Yes	Ambulatory	Zoster	Childhood in China
2	China-8	M	30	Yes	Ambulatory	Zoster	Childhood in China
2	China-9	F	67	Yes	Ambulatory	Zoster	Childhood in China
2	China-10	M	71	Yes	Ambulatory	Zoster	Not available
2	China-11	M	74	Yes	Hospitalized	Zoster	Childhood in China
2	China-12	F	80	Yes	Hospitalized	Zoster	Not available
2	China-13	F	40	Yes	Ambulatory	Zoster	Childhood in China
2	China-14	M	18	Yes	Ambulatory	Varicella	Not available
2	China-15	F	76	Yes	Ambulatory	Zoster	Not available
2	China-16	M	47	Yes	Ambulatory	Zoster	Childhood in China
2	China-17	M	20	Yes	Ambulatory	Zoster	Childhood in China
2	China-18	M	17	Yes	Ambulatory	Varicella	Not available
2	China-19	F	32	Yes	Ambulatory	Zoster	Childhood in China
2	China-20	F	12	No	Ambulatory	Varicella	Not available
2	China-21	M	78	No	Hospitalized	Zoster	Childhood in China
2	China-22	F	86	Yes	Hospitalized	Zoster	Childhood in China
2	China-23	F	23	Yes	Ambulatory	Varicella	Not available
2	China-24	M	24	Yes	Ambulatory	Zoster	Childhood in China
2	China-25	M	16	Yes	Ambulatory	Varicella	Not available
2	China-26	F	56	Yes	Ambulatory	Zoster	Childhood in China
2	China-27	F	68	Yes	Ambulatory	Zoster	Childhood in China
2	China-28	F	32	Yes	Ambulatory	Zoster	Childhood in China
2	China-29	F	46	Yes	Ambulatory	Zoster	Childhood in China
2	China-30	F	9	Yes	Ambulatory	Varicella	Not available
2	China-31	M	58	Yes	Ambulatory	Varicella	Not available
2	China-32	M	69	Yes	Ambulatory	Zoster	Childhood in China
2	China-33	F	71	Yes	Hospitalized	Zoster	Childhood in China
2	China-34	M	21	Yes	Ambulatory	Zoster	Childhood in China
2	China-35	M	3	Yes	Ambulatory	Zoster	Childhood in China
2	China-36	M	10	Yes	Ambulatory	Zoster	Childhood in China
2	China-37	M	25	Yes	Ambulatory	Zoster	Childhood in China
2	China-38	F	31	Yes	Ambulatory	Zoster	Childhood in China
2	China-39	F	26	Yes	Ambulatory	Zoster	Childhood in China
2	China-40	M	37	Yes	Ambulatory	Zoster	Childhood in China
2	China-41	F	28	Yes	Ambulatory	Zoster	Childhood in China
2	China-42	F	51	Yes	Ambulatory	Zoster	Childhood in China
2	China-43	F	50	Yes	Ambulatory	Zoster	Childhood in China
2	China-44	M	26	Yes	Ambulatory	Zoster	Childhood in China
